# Commonalities for comorbidity: Overlapping features of the endocannabinoid system in depression and epilepsy

**DOI:** 10.3389/fpsyt.2022.1041460

**Published:** 2022-10-19

**Authors:** S. Alisha Epps

**Affiliations:** Department of Psychology, Whitworth University, Spokane, WA, United States

**Keywords:** depression, epilepsy, comorbidity, endocannabinoid system, anandamide, 2-arachidonoyl glycerol, cannabinoid receptors, fatty acid amino hydrolase

## Abstract

A wealth of clinical and pre-clinical data supports a bidirectional comorbidity between depression and epilepsy. This suggests commonalities in underlying mechanisms that may serve as targets for more effective treatment strategies. Unfortunately, many patients with this comorbidity are highly refractory to current treatment strategies, while others experience a worsening of one arm of the comorbidity when treating the other arm. This highlights the need for novel pharmaceutical targets that may provide safe and effective relief for both depression and epilepsy symptoms. The endocannabinoid system (ECS) of the brain has become an area of intense interest for possible roles in depression and epilepsy. Several existing literature reviews have provided in-depth analysis of the involvement of various aspects of the ECS in depression or epilepsy separately, while others have addressed the effectiveness of different treatment strategies targeting the ECS in either condition individually. However, there is not currently a review that considers the ECS when both conditions are comorbid. This mini-review will address areas of common overlap between the ECS in depression and in epilepsy, such as commonalities in endocannabinoids themselves, their receptors, and degradative enzymes. These areas of overlap will be discussed alongside their implications for treatment of this challenging comorbidity.

## Introduction

A bidirectional comorbidity exists between epilepsy and depression, such that patients with one condition are at a statistically elevated risk of developing the other. Clinically, anywhere from 22.8% (1) to 50% (2) of patients with epilepsy also have comorbid depression. Likewise, patients with depression have a 1.7- to 4.2-fold greater risk of epilepsy than the general population (3). Despite the prevalence of this comorbidity, it remains challenging to treat, with high rates of pharmacoresistance and worsened progression of illness (4). These challenges have driven the search for new treatments that are safe and effective for treating comorbid epilepsy and depression, with the endocannabinoid system (ECS) providing a recent target of interest in preclinical models.

A number of excellent reviews have discussed the ECS and its putative role in either depression or epilepsy [for examples, see (5–8)]. This mini-review will not seek to exhaustively review each condition but will instead focus on areas of commonality in the ECS between depression and epilepsy. Briefly, the ECS of the brain involves two key compounds, anandamide (AEA) and 2-arachidonoyl glycerol (2-AG). These compounds bind with varying degrees of efficacy to two key types of cannabinoid receptors, CB1 and CB2. Both are G-protein coupled receptors that may then exert effects on other neurotransmitters, such as dopamine, serotonin, glutamate, and GABA. AEA is enzymatically degraded by fatty acid amino hydrolase (FAAH), while 2-AG is degraded by monoacylglycerol lipase (MAGL) (9).

## Components of the endocannabinoid system in depression and epilepsy

### Anandamide (AEA)

Increased activity of AEA is associated with antidepressant-like effects. This has been demonstrated by administration of AEA itself (10), known antidepressants (11–13), uptake inhibitors (14), and similar compounds like oleoylethanolamide (15). However, it should be noted that pre-clinical models and clinical examples of depression demonstrate both decreases (16–18) and increases in AEA within the brain (18, 19). While this might initially seem contradictory, regional specificity of effects and differences between models may be vital to reconciling these findings. Within the nucleus accumbens, AEA was increased in the olfactory bulbectomized model of depression (18). The striatum of both olfactory bulbectomized and Wistar-Kyoto rats reflect decreases in AEA (18), while serum levels of AEA are generally increased in both patients with mild depression (20) and Flinders Sensitive Rats [(19), but see also (17) for a decrease]. The prefrontal cortex showed decreases in AEA in the olfactory bulbectomized rats but increases in the Wistar-Kyoto model (18). The hippocampus exhibited decreases in AEA in the olfactory bulbectomized model (18), chronic unpredictable mild stress (16), and social isolation models (21), but different effects based on lateralization in the Flinders Sensitive rat (19). While none of these studies directly addressed comorbidity with epilepsy, it is of interest to note that the olfactory bulbectomy model of depression exhibits heightened susceptibility to seizures (22); thus, the decreases it exhibits in AEA in all assessed regions except the nucleus accumbens may be of particular relevance to the comorbidity.

AEA was generally found to have a similar role in epilepsy and seizure susceptibility, with increases in AEA generally being anticonvulsant against slow wave discharges (23) or providing a compensatory mechanism to protect the brain against excess glutamate release during a seizure (24–26). AEA did, however, show differing effects in some aspects of epilepsy, with Rocha et al. (27) uncovering increased cortical AEA in mesial temporal lobe epilepsy (MTLE) of humans, while Colangeli et al. (28) and Romigi et al. (29) showed decreases in AEA in amygdala kindling and the cerebrospinal fluid of patients with TLE, respectively.

Taken together, these findings suggest that increasing AEA may be generally antidepressant and anticonvulsant, though care should be taken to consider the relevance of different brain regions and different models to the comorbidity of depression and epilepsy.

### 2-Arachidonoyl glycerol (2-AG)

Compared to AEA, 2-AG has been less well–researched, particularly regarding epilepsy. Those findings that do exist for treatment strategies of depression appear in many ways consistent with those of AEA. Increases in 2-AG brought on by chronic antidepressants (11, 30) or repetitive transcranial magnetic stimulation (31) generally appeared beneficial. However, rodent models of depression often showed changes in 2-AG different from those seen with AEA, making the interpretation of increases in 2-AG less straightforward than a clear antidepressant effect. For example, olfactory bulbectomized rats showed a decrease in 2-AG in the nucleus accumbens and an increase in the prefrontal cortex (PFC) (18), both of which were opposite in direction compared to the changes in AEA seen in this model. Additionally, the Flinders Sensitive Line showed a decrease in 2-AG in the PFC (19), in contrast to the olfactory bulbectomy model. Interestingly, two different models of unpredictable chronic mild stress and Flinders Sensitive Line all showed decreases in 2-AG in the hippocampus (19, 32, 33), suggesting an overall trend toward decreased 2-AG in rodent models of depression.

2-AG is largely understudied in epilepsy, with one study of absence seizures in Genetic Absence Epilepsy Rat from Strasbourg (GAERS) reporting an increase in 2-AG (34) and another study in patients with MTLE reporting a decrease (27). Taken together, it seems that 2-AG may be involved in depression- and epilepsy-comorbidity, though it should also be noted that several studies of each condition showed no changes in 2-AG levels [for example, (28, 35, 36)]. Agents that increase 2-AG may be helpful for depression, but much more work is needed to assess their safety and/or efficacy as a treatment strategy in epilepsy.

### Cannabinoid 1 receptor (CB1)

The CB1 receptor is the most extensively researched aspect of the ECS in both depression and epilepsy individually, likely due to its high prevalence in brain regions relevant to each condition. In regard to depression, CB1 agonists (14, 37–39) or agents that increased CB1 binding or receptor density (16, 18, 30, 40–42) were generally antidepressant. Conversely, CB1 antagonists or knockout of the CB1 gene often blocked the effects of known antidepressants (10, 14, 37, 38, 42, 43). CB1 knockout alone increased emotional behaviors (44) with downstream serotonergic (45) and hippocampal effects (46). However, there were several examples of CB1 antagonists enhancing antidepressant effects (32, 47–49), or of known antidepressants resulting in a decrease in CB1 receptor binding properties (30, 50, 51). These differences may be reconciled by localization of the CB1 receptor on both glutamatergic and GABAergic neurons, with changes to CB1 receptors on glutamatergic neurons having different downstream effects than those on GABAergic neurons (24, 52, 53). These findings could also be due to compensatory changes and downregulation resulting from chronic CB1 receptor activation. Regardless, existing evidence generally supports elevated CB1 function as antidepressant, while decreases in CB1 function may be either pro- or anti-depressant.

This complexity is mirrored by the role of CB1 in epilepsy. There are a multitude of examples in which increases in CB1 function have a protective effect against ear clip seizure (54), slow wave discharges (34, 55, 56), hyperthermia-induced seizure (57), pilocarpine models of MTLE (58, 59), or audiogenic seizures (60). Other studies have demonstrated an increase in CB1 receptor expression following seizure in temporal lobe kindling (61, 62), some brain regions of GASH/Sal hamsters (63), and pilocarpine models of MTLE (64, 65), or have shown a detrimental role of CB1 agonists worsening generalized seizure activity (66, 67). Thus, it appears that involvement of the CB1 receptor in epilepsy may depend on the type of seizure (focal vs. generalized), timeline of progression (acute vs. chronic), and type of agent used (agonist vs. modulator). However, CB1 receptor antagonists or genetic ablations are overwhelmingly pro-convulsant in models of temporal lobe (24, 53, 59) and generalized epilepsies (34, 56, 57, 68, 69).

Despite the wealth of research supporting a role of CB1 receptors in depression and epilepsy individually, little has been done to assess CB1 function when both conditions are comorbid. The presence of studies supporting an antidepressant role and an anticonvulsant role for agents increasing CB1 function suggests they may be effective therapeutic agents for the comorbidity, but future studies must exercise extreme care that these agents remain safe for both arms of the comorbidity.

### Cannabinoid 2 receptor (CB2)

Unlike the CB1 receptor, CB2 has been much less studied regarding depression and epilepsy. This may be due to its lower expression within the brain relative to CB1 (9). When studied, increases in CB2 function by agonists (37, 39, 70) generally alleviate depression-related symptoms. However, the same can be said for antagonists or inverse agonists (37, 48) and known antidepressants that decrease CB2 function (18). Rodent models of depression show similarly incongruent changes; the olfactory bulbectomized rat shows a decrease in CB2 expression in the PFC and hippocampus, while the Wistar-Kyoto rat shows an increase in CB2 levels in the dorsal striatum and cerebellum (18).

The majority of studies of epilepsy to date suggest that any change in CB2 receptor properties can result in either no effect or a potentially detrimental effect. Increases in CB2 receptor signaling or expression were seen in cortical cells from patients with MTLE (71) and patients with Dravet syndrome (72). However, patients with MTLE also showed decreased CB2 signaling in the hippocampus (71), and antagonism or knockout of CB2 receptors was associated with worsening of seizures in multiple models like pentylenetetrazole and 6-Hz induced seizures [(73, 74), although the latter showed no effect on flurothyl or kainic acid induced seizures]. It is possible that some of the increases in CB2 receptor function seen in patients with MTLE and Dravet syndrome may be compensatory, thereby reconciling these findings. Regardless, the complexity of the putative role for CB2 in depression or epilepsy warrants careful consideration when approaching agonists or antagonists of this receptor as possible treatment strategies.

### Fatty acid amino hydrolase (FAAH)

The degradatory enzyme FAAH has also been targeted for treating depression and epilepsy individually, with the general hypothesis that inhibiting or decreasing FAAH would increase levels of AEA, thereby alleviating symptoms. Indeed, increases in FAAH have been seen in both the bulbectomized and Wistar-Kyoto models of depression (18, 75), suggesting an overabundance of degradation of AEA may be responsible for depression symptomatology. In humans, the A allele of the FAAH C385A SNP was associated with higher depression score (76). However, there are examples of other models of depression in which FAAH is not involved (16, 77), or in which antidepressants decrease the amount of FAAH (78), making this a less clear association.

Treatment with FAAH inhibitors like URB-597 are nearly always antidepressant, strengthening the idea that this may be an effective target for alleviating depression symptoms. FAAH inhibitors have demonstrated effectiveness in alleviating depression-related behavior in the Wistar-Kyoto (75), chronic unpredictable stress (79), social isolation (21), early life stress (80), and Forced Swim Test (14) models. There were, however, other studies that reported no effect of URB-597 on emotional responses (38, 81), and decreases in FAAH were also present in certain brain regions in animal models of depression like the Wistar-Kyoto (18) and Flinders Sensitive Line (19).

Similarly, treatment with FAAH inhibitors is largely anticonvulsant, though these studies have largely been focused on models of TLE. FAAH inhibitors were shown to decrease severity of seizure (26) and cellular damage (82) in a kainic acid model of TLE, and also improved susceptibility of the dentate gyrus to electrical stimulation and pilocarpine-induced seizure (83). While Wendt et al. reported no effects of FAAH inhibition on amygdala kindling behaviors (84), other studies have reported a restoration of inhibitory/excitatory balance in the same model (28) and a protective effect against seizures induced by ear clip electrodes (54).

Taken together, most studies with FAAH inhibitors suggest a beneficial effect on depression- and epilepsy-related symptoms when studied separately, though there are exceptions. Importantly, studies that lacked a beneficial effect of FAAH inhibition did not show a detrimental effect, suggesting that this may be a safe treatment option to consider in addressing the comorbidity. It is also interesting to note that FAAH inhibitors were often effective even in models that did not show a baseline impairment to FAAH, such as the chronic unpredictable stress model (16, 79). It is possible that methodological differences between labs are involved; however, it may be of interest for future studies to assess both baseline FAAH and response to FAAH inhibitors in the same model, as this may broaden our understanding of the role of FAAH and its therapeutic potential.

### Monacylglycerol lipase (MAGL)

Like FAAH, MAGL is also a degradatory enzyme. Unlike FAAH, it is responsible for the hydrolysis of 2-AG and is comparatively understudied in depression and epilepsy. Despite that, increases in MAGL levels or activity have been observed in some regions of the olfactory bulbectomized (18), Wistar-Kyoto (18), and chronic restraint stress (85) models of depression, suggesting that elevations in MAGL may evoke depression-related symptoms by increased hydrolysis of 2-AG. As would be expected from this hypothesis, treatment with MAGL inhibitors was protective against depression from early life stress (80) and chronic mild stress (33).

However, other models and brain regions suggest that a *decrease* in MAGL may be more closely associated with depression, as seen in the chronic mild stress (32) and Flinders Sensitive Line (19) models. As with other aspects of the ECS, region specificity may be crucial to understanding the role of MAGL in depression. Knockdown of MAGL specific to the habenula results in antidepressant effects (86), while MAGL inhibitor JZL184 decreased intracranial self-stimulation to the medial forebrain bundle (77).

Findings related to MAGL in epilepsy are conflicting. Anderson reported that MAGL inhibitors reduced seizures induced by hyperthermia but worsened spontaneous seizures (57). MAGL inhibitors were reported to improve kindling acquisition but to have no effect on fully kindled seizures (87). Postmortem samples from patients with TLE showed no changes in MAGL (52).

Given these results, MAGL seems less promising of a candidate for treating depression and epilepsy comorbidity than other aspects of the ECS. However, it has also been less well–studied than some of the other aspects, and thus may warrant further consideration before conclusions are fully drawn. Perhaps it is best suited only to specific subtypes of depression and epilepsy, but future studies would be needed to make this determination.

## Discussion

The endocannabinoid system has been recognized as a potential target for treating depression or epilepsy individually. AEA, the CB1 receptor, and FAAH are the most well-studied aspects of the ECS related to these conditions. Increasing the effects of AEA seems generally antidepressant and anticonvulsant, whether approached by targeting AEA itself, elevating function of CB1 receptors to bind AEA, or preventing degradation of AEA by inhibition of FAAH. Other aspects of the ECS, like 2-AG, CB2, and MAGL, remain understudied. The studies that do exist suggest CB2 and MAGL may need to be approached with caution for depression and epilepsy comorbidity, but 2-AG appears to hold some promise as a treatment strategy. Even for the best studied of these aspects, there are many complexities and nuances to this relationship that must be considered in future studies.

Novel treatment strategies must always be approached with the perspective of “first, do no harm” balanced alongside analysis of risks and benefits (88), and this is especially important for the comorbidity of depression and epilepsy. Treatments that benefit one arm of the comorbidity may have detrimental effects on the other arm, as with anticonvulsants that may result in depressed mood as a side effect (89). While this review has attempted to guide considerations for the comorbidity by identifying areas of overlap and commonality between studies of depression and epilepsy individually, very few studies to date have addressed the ECS in a true model of the comorbidity that assesses both conditions simultaneously. This is of utmost priority for identifying the role of endocannabinoids in comorbid depression and epilepsy and understanding the safety and efficacy of potential treatment strategies. Additionally, it is also of importance to consider the bidirectionality of this relationship. While many findings from models of epilepsy with depression-related behaviors are similar to those from models of depression that exhibit seizures, it cannot be said with certainty that both directions of this comorbidity are the same and will respond similarly to treatment. Thus, assessing the role and therapeutic efficacy of the ECS in both directions of the bidirectional comorbidity of depression and epilepsy is a key next step.

Additionally, there are complexities within the ECS itself that need to be considered in light of their therapeutic potential. Several of the reviewed studies have suggested that the role of the ECS in depression or epilepsy may be regionally or temporally specific. For example, models of depression like the Wistar-Kyoto and olfactory bulbectomized rat showed regional specificity in baseline levels of ECS parameters, with some regions showing an increase in a parameter and others showing a decrease (18). This is an important, albeit challenging, factor to consider regarding treatment, where all regions are typically affected similarly. Likewise, models of epilepsy have shown temporal specificity in regard to the stage of epileptogenesis when a treatment is effective, such as MAGL inhibitors that improve seizure parameters only during acquisition of kindling but have no effect once the fully kindled state is reached (87). This may suggest the presence of a critical window during which treatment must be initiated and should be addressed further in future studies of both pediatric and adult epilepsies. Some parameters of the ECS also have cellular specificity, such as CB1 receptors localized to both glutamatergic and GABAergic synapses. This localization may be crucial to understanding the development of the comorbidity and to reconciling differing results in studies of proconvulsant and anticonvulsant effects of CB1 modulation. Lastly, the presence and role of compensatory changes to the ECS are worth consideration as well. Several studies of both human and animal models have suggested increases in CB1 receptor function that develops as a compensatory response to altered neurotransmitter states and inhibitory/excitatory balance of depression or epilepsy. The role of these compensatory changes in function of the ECS should be addressed to better understand their impact on treatment effects.

In conclusion, commonalities in roles of endocannabinoids, their receptors, and degradative enzymes between depression and epilepsy when studied individually support study of these components in comorbid depression and epilepsy. The ECS may be an important target for safe and effective treatment strategies, although many important complexities of this relationship remain for further study.

## Author contributions

SAE confirms sole responsibility for literature review, interpretation, and drafting and revision of the article.

## Funding

This research was supported by a grant from the M. J. Murdock Charitable Trust (NS-201914142) and funding from the Whitworth University psychology department. Each of these funders will contribute toward the cost of open access publication fees.

## Conflict of interest

The author declares that the research was conducted in the absence of any commercial or financial relationships that could be construed as a potential conflict of interest.

## Publisher's note

All claims expressed in this article are solely those of the authors and do not necessarily represent those of their affiliated organizations, or those of the publisher, the editors and the reviewers. Any product that may be evaluated in this article, or claim that may be made by its manufacturer, is not guaranteed or endorsed by the publisher.
